# Imaging characteristics and diagnostic accuracy of FDG-PET/CT, contrast enhanced CT and combined imaging in patients with suspected mycotic or inflammatory abdominal aortic aneurysms

**DOI:** 10.1371/journal.pone.0272772

**Published:** 2022-08-09

**Authors:** Lars Husmann, Martin W. Huellner, Hannes Gruenig, Bruno Ledergerber, Michael Messerli, Carlos-A. Mestres, Zoran Rancic, Barbara Hasse

**Affiliations:** 1 Department of Nuclear Medicine, University Hospital Zurich, University of Zurich, Zurich, Switzerland; 2 Division of Infectious Diseases and Hospital Epidemiology, University Hospital Zurich, University of Zurich, Zurich, Switzerland; 3 Clinic for Cardiac Surgery, University Hospital Zurich, University of Zurich, Zurich, Switzerland; 4 Department of Vascular Surgery, University Hospital Zurich, University of Zurich, Zurich, Switzerland; ARNAS Civico Di Cristina Benfratelli: Azienda Ospedaliera di Rilievo Nazionale e di Alta Specializzazione Civico Di Cristina Benfratelli, ITALY

## Abstract

**Purpose:**

To evaluate the diagnostic accuracy and specific imaging characteristics of positron emission tomography/computed tomography with 18F-fluorodeoxyglucose (PET/CT), contrast enhanced CT (CE-CT), and a combined imaging approach (CE-PET/CT) in patients with infectious/mycotic (MAA), inflammatory (IAA), and non-infected, non-inflammatory abdominal aortic aneurysm (AAA).

**Materials and methods:**

In this single-center retrospective cohort study, all imaging data sets of 29 consecutive patients with clinically suspected MAA or IAA were anonymised with different, reshuffled identification numbers and retrospectively and independently analysed by two experienced readers, blinded to all clinical patient data. Readers determined the presence or absence and MAA, IAA and AAA and of predefined imaging characteristics (e.g. fluid collection), and measured metabolic activity and wall thickness of all aneurysms. A multidisciplinary team of specialists served as standard of reference and re-evaluated every clinical case, considering all clinical, laboratory, microbiological, histopathological and imaging results, including all follow-up examinations.

**Results:**

Diagnostic accuracy was higher in PET/CT as compared to CE-CT in differentiating AAA from MAA and IAA: area under the receiver operating characteristic curve (AUC-ROC) *0*.*81 (95%* confidence intervals *0*.*69–0*.*92) and 0*.*63 (0*.*52–0*.*74) (P = 0*.*027)*. Specific imaging characteristics were significantly associated with different types of aneurysms (P<0.05), i.e. very high metabolic activity and dorsal sparing of metabolic activity in PET/CT and wall thickening in CE-CT were indicative for IAA; fat stranding and fluid collections in CE-CT were associated with MAA; while low metabolic acitivity and absence of wall thickening in PET/CT, and less fat stranding and absence of wall thickening in CE-CT were indicative for non-infected, non-inflammatory AAA.

**Conclusion:**

Specific imaging characteristics of PET/CT and CE-CT may be helpful in differentiating between MAA, IAA, and non-infected, non-inflammatory AAA.

## Introduction

Abdominal aortic aneurysms may occasionally be caused by infection or inflammation. Infectious/mycotic (MAA) aneurysms are reported with a prevalence of 0.7–4.5% of all aortic aneurysms [1], while inflammatory aneurysms (IAA) account for 5–25% of all abdominal aneurysms [2]. Management of MAA and IAA is diverging and early diagnosis is a cornerstone of effective treatment [2–4]. Differentiating MAA, IAA, and non-infected, non-inflammatory abdominal aortic aneurysm (AAA) is a challenging clinical problem [5].

Positron emission tomography/computed tomography with ^18^F-fluorodeoxyglucose (PET/CT) seems to be a sensitive and accurate imaging modality in the field of infection and inflammation, particularly in vascular diseases, such as arteritis [5] and vascular graft infections [6–8]. In imaging of abdominal aneurysms in particular, various imaging characteristics have been decribed for contrast enhanced computed tomography (CE-CT) [2, 9]. However, specific imaging characteristics for the discrimination of MAA, IAA as well as AAA are lacking, limiting the diagnostic accuracy of imaging [10].

Therefore, the aim of the present study was to evaluate the diagnostic accuracy and specific imaging characteristics of PET/CT, CE-CT, and a combined imaging approach (CE-PET/CT) in patients with MAA, IAA, and non-infected, non-inflammatory AAA.

## Materials and methods

### Study design and data collection

This retrospective study included consecutive patients, who were examined with PET/CT and contrast enhanced CT between the years 2005 and 2018 at the University Hospital of Zurich. Patients were screened, if the term “mycotic”, “infectious” or “inflammatory” aneurysm was mentioned in the PET/CT report, including the clinical data and question section. Patients were included into the study population, if an aneurysm of the abdominal or pelvic arteries was described in the report (regardless of the suspected status of inflammation or infection), and if both imaging modalities were performed within two months and prior to any vascular interventions (e.g., graft placement).

A multidisciplinary board of specialists (cardiac surgeons, vascular surgeons, specialists in infectious diseases, radiologists, nuclear medicine physicians and microbiologists) served as standard of reference. This board retrospectively re-evaluated every clinical case, considering all clinical, laboratory, microbiological, histopathological and imaging results, including all follow-up examinations.

The institutional review board approved the study (BASEC-Nr. 2018–01904), and we obtained written informed consent from all participants who were examined between the years 2016 and 2018. For patients scanned between the years 2005 and 2015, written informed consent was waived due to retrospective inclusion. All procedures were performed in accordance with the 1964 Helsinki declaration and its later amendments or comparable ethical standards.

### PET/CT and CE-CT data acquisition

All PET/CT and CE-CT examinations followed basic study protocols. For PET/CT, patients fasted for at least four hours, FDG dosage was body-weight adjusted, the uptake time was standardized to 60 minutes in supine position, a non-enhanced CT scan was performed and used for attenuation correction, and data was acquired with arms overhead whenever possible. Blood glucose levels <12 mmol/l were accepted. Body weight, height, and blood glucose level were measured prior to imaging. Five different types of PET/CT scanners were used throughout the study period, i.e. Discovery STE, Discovery LS, Discovery RX, Discovery MI, and Discovery 690 (all GE Healthcare, Waukesha, WI). To compensate for differences in the sensitivity of the different PET/CT scanner generations, we measured the metabolic activity in the mediastinal blood pool and in the liver tissue for reference.

For CE-CT of the abdomen, 80 ml iodinated contrast material (Visipaque® 320, GE Healthcare) were injected, timed for imaging at the portal venous phase with a tube voltage of 120 kV and a tube current–time product of 100–320 mAs. If patients had a recent CE-CT of the region of interest prior to the PET/CT, the CE-CT was not repeated.

### Image analysis

All PET/CT, CE-CT, and combined CE-PET/CT data sets were independently analysed by two experienced and double board certified radiologists and nuclear medicine physicians using a AW Workstation Version 4.6 (GE Healthcare Biosciences, Pittsburgh, PA). Readers were blinded to all clinical patient data. Data sets of each patient (PET/CT, CE-CT, combined CE-PET/CT) were anonymised, using three different, reshuffled identifications numbers, and a time frame of at least four weeks was set between readings of each data set.

For all three imaging data sets, readers determined whether the aneurysm was either non-inflammatory/non-infectious or inflammatory/infectious. If readers found an aneurysm to be inflammatory/infectious, they were additionally asked to determine whether it was infectious or inflammatory.

For PET/CT, readers furthermore measured the maximum standardized uptake value (SUV_max_) in all aneurysms and the mean standardized uptake value (SUV_mean_) in the liver and in the mediastinal bloodpool with the use of an in-built software. For the latter, a volume-of-interest (VOI) was placed on the site of interest, and the correct placement of the VOI was confirmed by the use of axial, coronal, and sagittal reformatted images in order to avoid partial-volume effects or signal spillover from neighboring organs. Ratios of SUV were calculated, i.e. “SUVratio BP” was defined as SUVmax in the aneurysm divided by SUVmean in the mediastinal bloodpool, and “SUVratio liver” was defined as SUVmax in the aneurysm divided by SUVmean in the liver.

For PET/CT and CE-CT, readers were asked to determine whether arterial wall thickening was present in the aneurysm (yes or no), and readers measured the maximum wall thickness of the aneurysm. Furthermore, readers determined whether “dorsal sparing” was present, i.e. if increased FDG-avidity or increased wall thickening was only present in the non-dorsal parts of the aneurysm.

For CE-CT, readers were additionally asked to document the presence or absence of the following imaging findings: fat stranding, fluid collection, and contrast enhancement.

### Statistical analyses

Variables were expressed as median and interquartile range (IQR; 25th, 75th percentiles) or percentages. Sensitivity, specificity, negative predictive values (NPV), positive predictive values (PPV) and accuracy were determined for the diagnosis of MAA, IAA, and AAA for both readers individually and combined for all imaging modalities (i.e. PET/CT, CE-CT, and combined CE-PET/CT). Measures were complemented with binomial 95% confidence intervals (CI). Relevant differences in diagnostic accuracy between the three imaging methods and between the two readers were analysed by comparing the area under the receiver operating characteristic curve (AUC-ROC). Differences in sensitivity and specificiy of the three methods in diagnosing AAA were compared using McNemar tests.

Interobserver agreement was tested using weighted κ tests and interpreted according to the suggestions by Landis and Koch [11].

Differences in frequency or extent of all imaging characteristics were determined using Wilcoxon signed-rank tests (for SUVmax, SUVratios, and wall thickening (in mm)) and 2-sided Fisher’s exact tests (for dorsal sparing, fat stranding, fluid collection, contrast enhancement, and wall thickening (yes/no)).

A P-value of ≤0.05 was considered to indicate statistical significance. Statistical analysis was performed using commercially available software (Stata/SE, Version 17.0, StataCorp, College Station, Texas).

## Results

### Patient population

Patient demographics are displayed in Table 1. Twenty-nine patients fulfilled the inclusion criteria for the study, and no patients were retrospectively excluded. PET/CT and CE-CT were successfully performed with diagnostic image quality in all patients within a median of -3 days (interquartile range (IQR) -5–7). PET/CT was performed after body weight adapted intravenous injection of a median of 332 Megabecquerel of FDG (IQR 281–420).

**Table 1:** Baseline characteristics.

	All	MAA	IAA	AAA
**Number of patients**, n (%)	29 (100)	11 (38)	9 (31)	9 (31)
**Age**, median years (IQR)	64 (55–85)	68 (62–85)	62 (54–78)	69 (55–80)
**Male gender**, n (%)	25 (86)	8 (73)	9 (100)	8 (89)
**Diabetes mellitus**, n (%)	7 (24)	3 (27)	3 (33)	1 (11)
**White blood cell count**, median g/L (IQR)	9.4 (7.2–19.5)	10.6 (8.5–19.5)	9.1 (6.3–19.1)	8.8 (7.6–18.8)
**C-reactive protein**, median mg/L (IQR)	44 (14–234)	113 (49–234)	44 (9–107)	15 (13–137)
**Size of aneurysm**, mm (IQR)	56 (46–95)	61 (47–81)	50 (36–61)	61 (56–95)
**Location of aneurysm**				
**suprarenal**, n (%)	11 (40)	6 (55)	0 (0)	5 (56)
**juxtarenal**, n (%)	4 (14)	1 (9)	2 (22)	1 (11)
**infrarenal**, n (%)	12 (41)	4 (36)	5 (56)	3 (33)
**iliac**, n (%)	2 (7)	0 (0)	2 (22)	0 (0)

MAA, mycotic aortic aneurysm; IAA, inflammatory aortic aneurysm; AAA, abdominal aortic aneurysm; IQR, interquartile range

Eleven patients (38%) had a MAA, nine patients (31%) had an IAA, and nine patients (31%) had a non-infected, non-inflammatory AAA.

MAA were caused by *Escherichia coli*, *Staphylococcus aureus*, *Salmonella enteritidis*, *Streptococcus pneumoniae (2x)*, *Coxiella burnetii (2x)*, *Listeria monocytogenes*, *Porphyromonas gingivalis*, *Candida tropicalis* and *Mycobacterium bov*is, as confirmed by blood culture (6x), biopsy (2x), or tissue samples obtained during surgery (3x). Of nine patients diagnosed with IAA, three were associated with Ormond disease and one patient had a chronic periaortitis. AAA were confirmed by clinical follow-up without antibiotic or anti-inflammatory treatment in 8/9 patients (median follow-up 1523 days (894–3017)), one patient was lost to follow-up.

### Diagnostic performance

**Fig 1:**
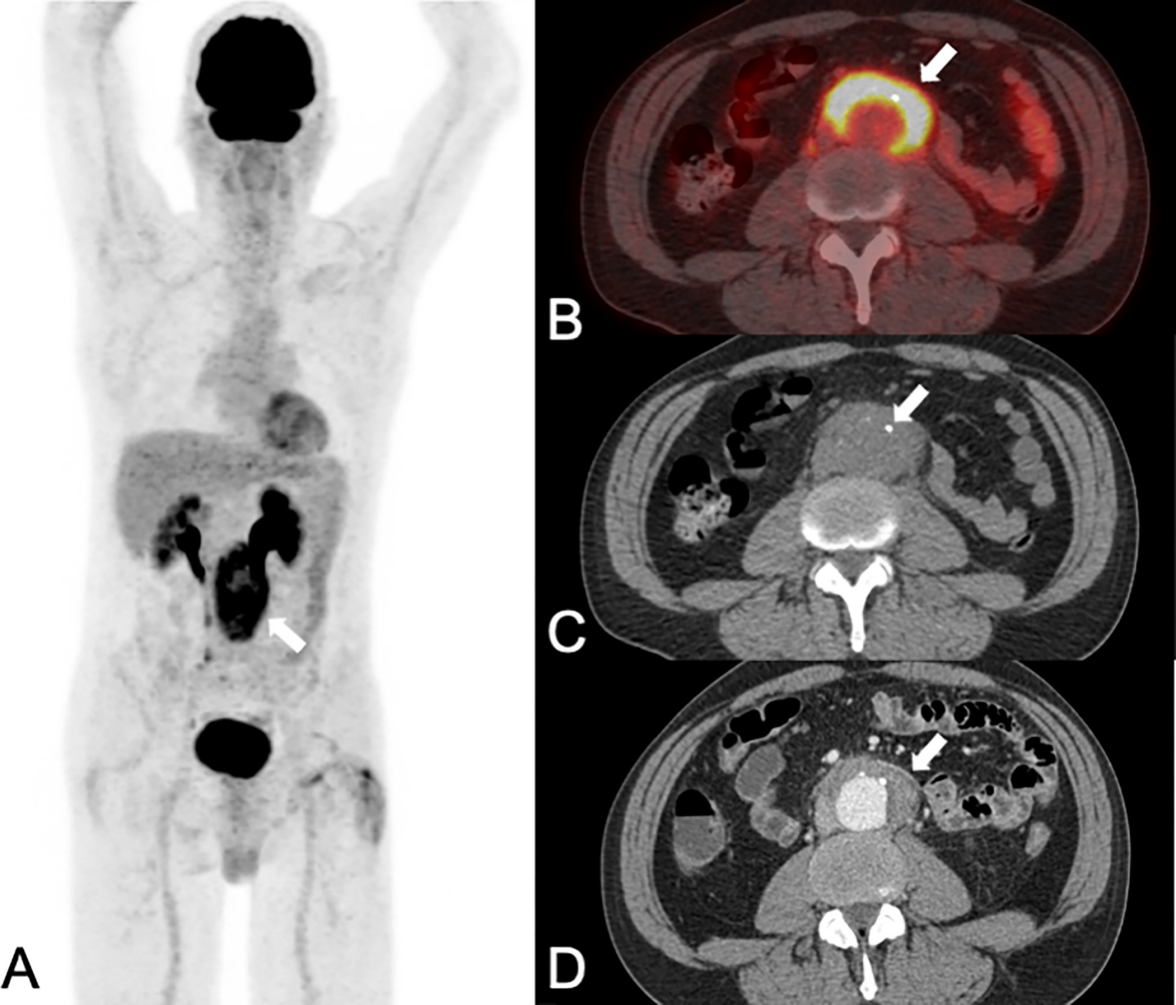
A 58-year-old man with an immunoglobulin G4-associated inflammatory abdominal aortic aneurysm (Ormond disease), initially presented with back pain and fever. PET/CT (A: maximum intensity projection of PET; B: fused PET/CT images; C: native CT images) and contrast-enhanced CT (CE-CT: D) showed a 64 mm large abdominal aortic aneurysm (white arrows in A-D) with intense FDG uptake (SUVmax 9.0 in A and B) in the thickened (12 mm) aortic wall (white arrows in B-D) with prominent dorsal sparing. Using PET/CT images alone one reader correctly diagnosed an inflammatory aneurysm, while the other one falsely diagnosed an infectious/mycotic aneurysm. With CE-CT and with combined imaging both readers correctly diagnosed an inflammatory aneurysm. The patient was subsequently treated with percutaneous endovascular abdominal aortic aneurysm repair and steroid therapy. The latest follow-up imaging 2.5 years after the initial diagnosis (not shown), showed a smaller (42 mm) aneurysm with less FDG uptake (SUVmax 4.1); and the patient had no symptoms.

**Fig 2:**
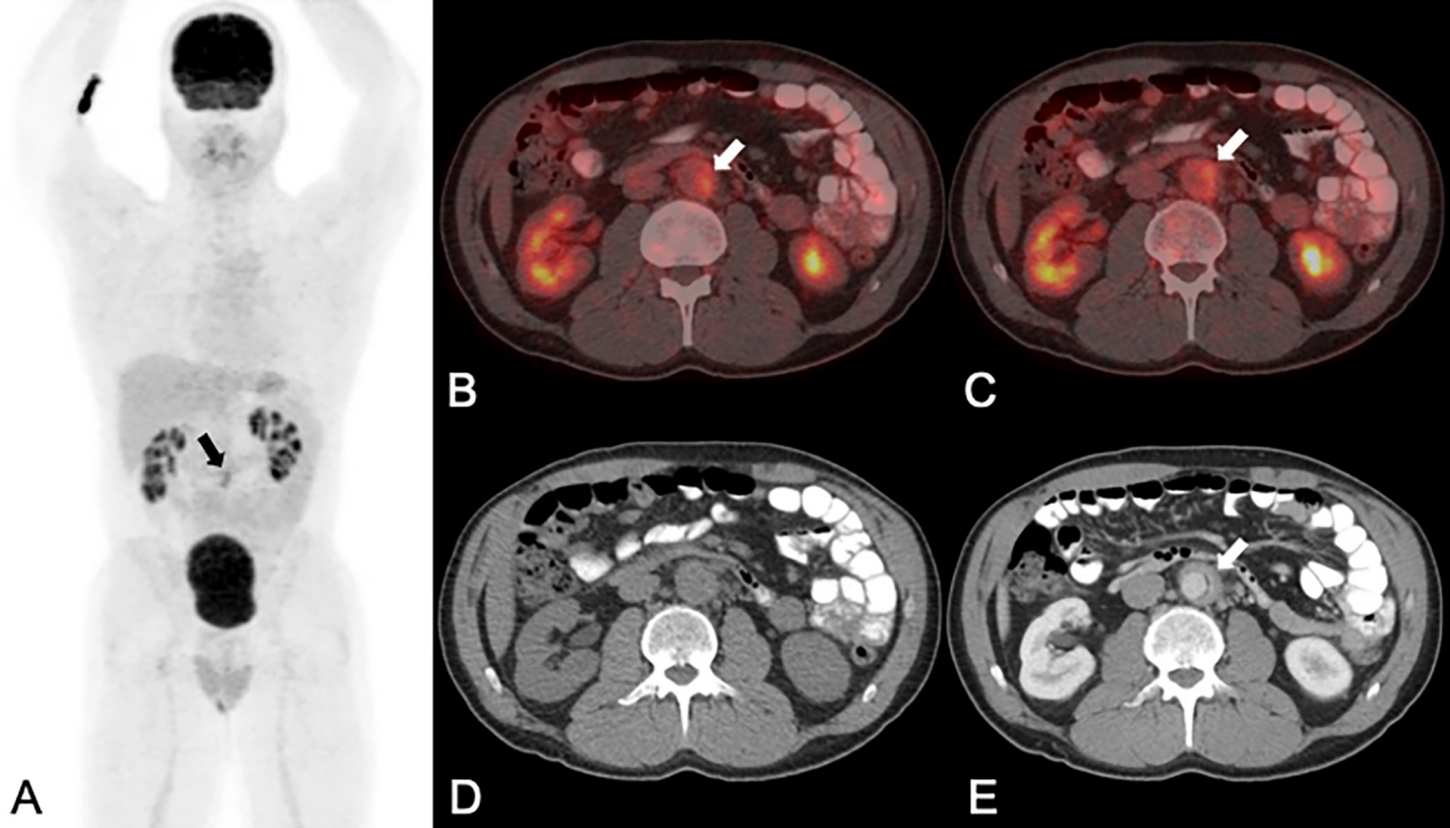
A 42-year-old man with chronic periaortitis, initially presenting with left-sided abdominal pain. PET/CT (A: maximum intensity projection of PET; B and C: fused PET/CT images (B) and contrast-enhanced CT (CE-CT: E) showed a 32 mm large abdominal aortic aneurysm (white arrows in B, C and E) with increased FDG uptake (SUVmax 3.5 in A-C) in the thickened (10 mm) aortic wall (white arrow in E). Using PET/CT images alone (A-D) both readers suspected an infectious/mycotic aneurysm (notably, wall thickening may not be clearly delineated on the non-enhanced CT part of the PET/CT in D, but only on the CE-CT in E). With CE-CT (E) and with combined imaging (not shown) both readers correctly diagnosed an inflammatory aneurysm. The patient was subsequently treated with steroid therapy for four months and no vascular intervention was performed. The latest follow-up imaging eight years after the initial diagnosis (not shown), showed no residual wall thickening in the normal sized (18 mm) abdominal aorta and the patient had no abdominal symptoms.

**Fig 3:**
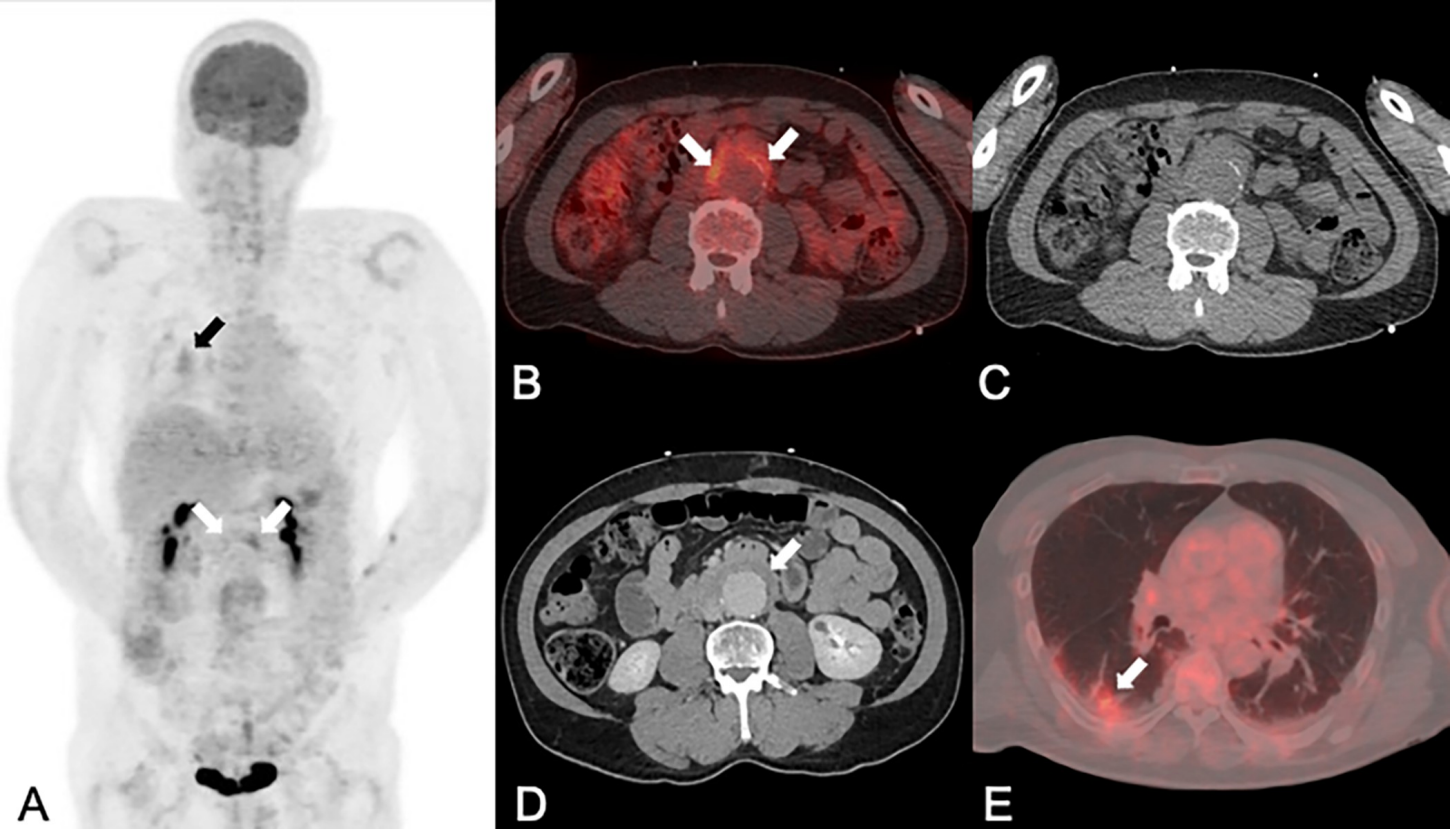
A 51-year-old man with an inflammatory abdominal aortic aneurysm, initially presenting with back pain and abdominal pain. PET/CT (A: maximum intensity projection of PET; B and E: fused PET/CT images) and contrast-enhanced CT (CE-CT: D) showed a 52 mm large abdominal aortic aneurysm (white arrows in B and D) with increased FDG uptake (SUVmax 3.6 in A and B) in the thickened (12 mm) aortic wall (white arrow in D) with dorsal sparing. Using PET/CT images alone (A-C) one reader correctly diagnosed an inflammatory aneurysm, while the other one falsely diagnosed an infectious/mycotic aneurysm (notably, wall thickening may not be clearly delineated on the non-enhanced CT part of the PET/CT in C, but only on the CE-CT in D). With CE-CT (E) and with combined imaging (not shown) both readers correctly diagnosed an inflammatory aneurysm. As an incidental finding, a small metabolically active pneumonia was diagnosed on PET/CT images (E). The patient was subsequently treated with percutaneous endovascular abdominal aortic aneurysm repair and steroid therapy. The latest follow-up imaging five years after the initial diagnosis (not shown), showed a stable abdominal aortic graft with no relevant wall thickening, and the patient had no abdominal symptoms.

**Fig 4:**
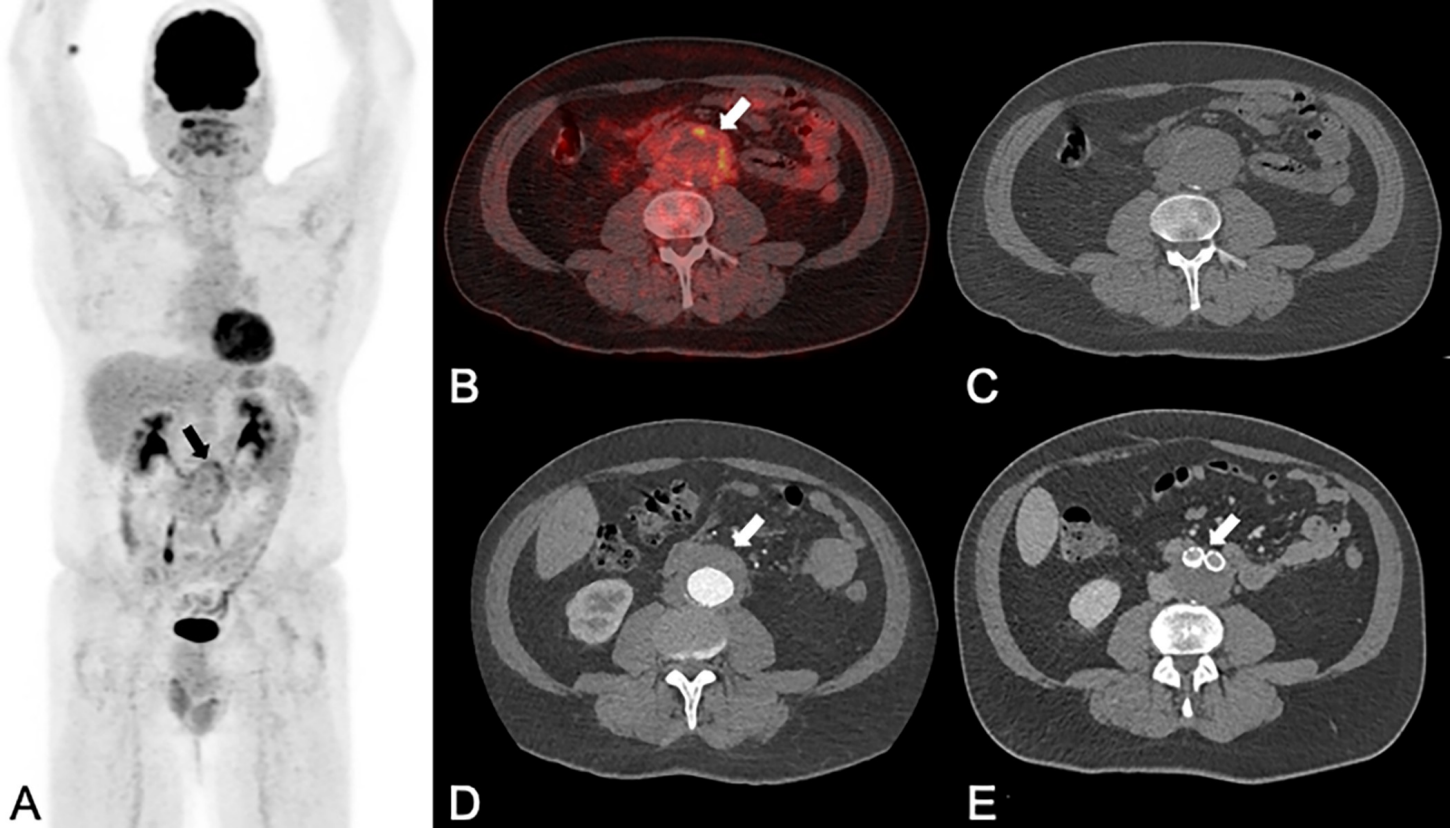
A 48-year-old man with a non-infected, non-inflammatory abdominal aortic aneurysm, initially diagnosed as an incidental finding on abdominal ultrasound. PET/CT (A: maximum intensity projection of PET; B: fused PET/CT images (B); C native CT images) and contrast-enhanced CT (CE-CT: D) showed a 55 mm large abdominal aortic aneurysm (white arrows in B and D) with increased FDG uptake (SUVmax 5.2, black arrow in A, white arrow in B) in the thickened (20 mm) aortic wall (white arrow in D). Both readers rated all imaging data set falsely: using PET/CT images and CE-CT images alone, one reader diagnosed a mycotic/infectious aneurysm while the other one diagnosed an inflammatory aneurysm; both readers suspected an inflammatory aneurysm on combined imaging. The patient was subsequently treated with percutaneous endovascular abdominal aortic aneurysm repair and neither antibiotic nor steroid therapy. The latest follow-up imaging four years after the initial diagnosis, showed no residual wall thickening abdominal aorta (E) and the patient had no abdominal symptoms.

**Fig 5:**
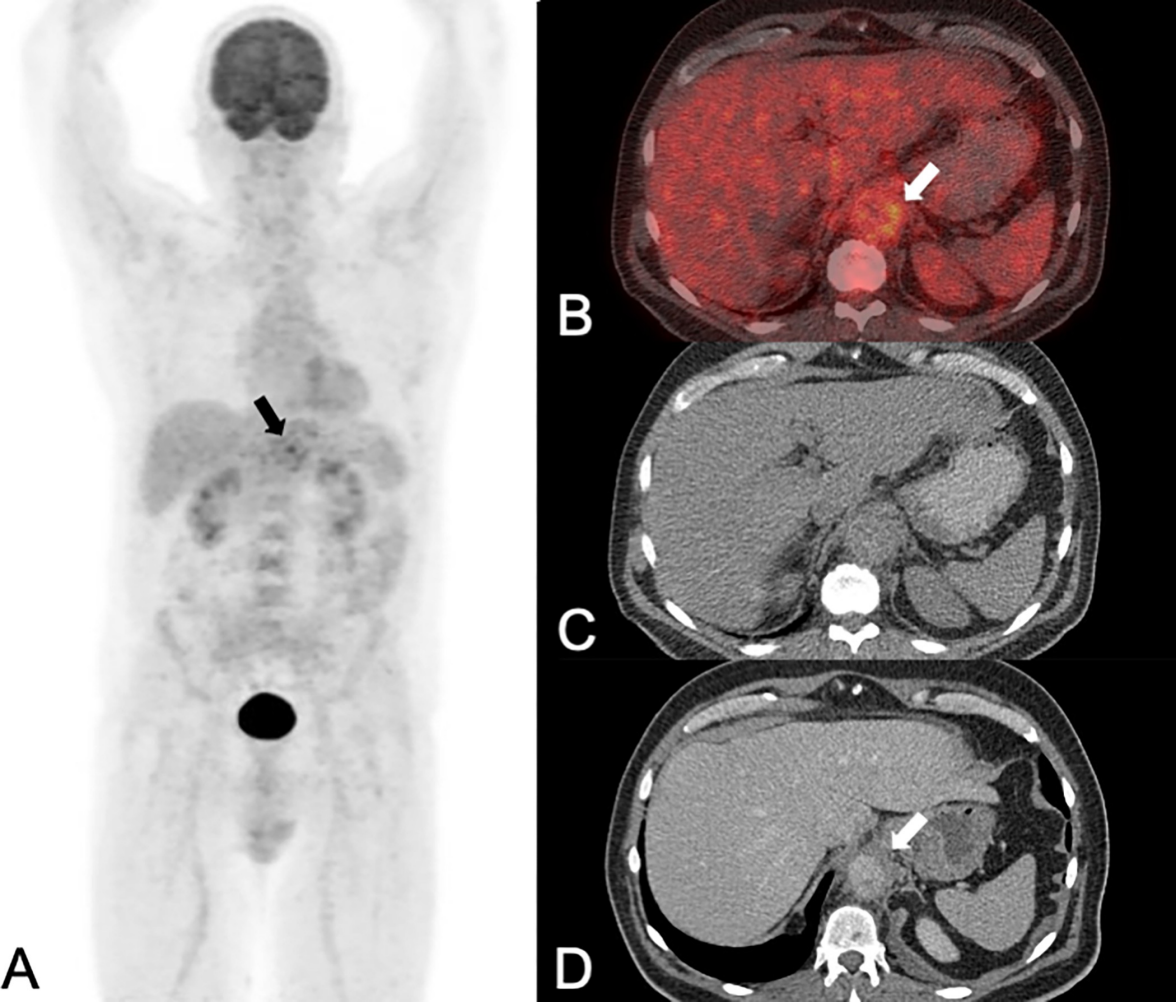
A 52-year-old man with an infectious/mycotic abdominal aortic aneurysm caused by *Streptococcus pneumoniae*, initially presented with abdominal discomfort and elevated C-reactive protein levels. PET/CT (A: maximum intensity projection of PET; B: fused PET/CT images; C native CT images) and contrast-enhanced CT (CE-CT: D) showed a 49 mm large abdominal aortic aneurysm (white arrows in B and D) with increased FDG-uptake (SUVmax 4.2; black arrow in A, white arrow in B) in the thickened (12 mm) aortic wall (white arrow in D). With all imaging data sets one reader correctly diagnosed an infectious/mycotic aneurysm, while the other one falsely diagnosed an inflammatory aneurysm on all imaging data sets. The patient was subsequently treated with endovascular aortic repair and antibiotic therapy.

Data for diagnostic accuracy of PET/CT, CE-CT, and combined CE-PET/CT for diagnosis of MAA, IAA and AAA for both readers combined are displayed in Table 2. A trend (P = 0.056) toward relevant differences in diagnostic accuracy between the three methods in diagnosing AAA was observed, with the lowest value of AUC-ROC (area under the receiver operating characteristic curve) for CE-CT and the highest value for PET/CT (Table 2). These differences were due to better sensitivities of PET images (61.1 vs. 27.8, p = 0.014), whereas specificities did not differ significantly (p > 0.3) (Table 2). P-values for the pairwise comparisons are: PET/CT vs. CE-CT P = 0.027; CE-CT vs. CE-PET/CT P = 0.064; PET/CT vs. CE-PET/CT P = 0.80. Kappa statistics for interrater agreement varied between “slight” and “substantial” agreement **(Figs 1–5;** Table 2; individual data for both readers is given S1 Table). Trends toward relevant differences of diagnostic accuracy between readers with respect to the three different diagnoses of MAA were observed (P = 0.078), IAA (P = 0.051), and AAA (P = 0.364), i. e., AUC-ROC (95% CI) for reader 1 was 0.639 (0.534–0.747) for MAA, 0.572 (0.460–0.684) for IAA, and 0.706 (0.607–0.804) for AAA; respective values for reader 2 were, 0.764 (0.672–0.856), 0.725 (0.620–0.829) and 0.769 (0.673–0.866) (Figs 1–5).

**Table 2:** Diagnostic accuracy of PET/CT, CE-CT, combined CE-PET/CT for diagnosis of MAA, IAA and AAA—combined for both readers, and Kappa statistics of the interrater agreement.

		Kappa	Sensitivity	Specificity	NPV	PPV	Accuracy	AUC-ROC	P*
								Area (95% CI)	
			% (95% CI)	% (95% CI)	% (95% CI)	% (95% CI)	% (95% CI)	(95% CI)	
**MAA**	PET/CT	0.198	72.7 (49.8–89.3)	55.6 (38.1–72.1)	76.9 (56.4–91.0)	50.0 (31.9–68.1)	62.1 (48.4–74.5)	0.641 (0.516–0.767)	0.282
	CE-CT	0.445	68.2 (45.1–86.1)	69.4 (51.9–83.7)	78.1 (60.0–90.7)	57.7 (36.9–76.6)	68.9 (55.5–80.5)	0.688 (0.563–0.814)	
	CE-PET/CT	0.368	77.3 (54.6–92.2)	77.8 (60.8–89.9)	84.8 (68.1–94.9)	68.0 (46.5–85.1)	77.6 (64.7–87.5)	0.775 (0.662–0.888)	
**IAA**	PET/CT	0.048	33.3 (13.3–59.0)	77.5 (61.5–89.2)	72.1 (56.3–84.7)	40.0 (16.3–67.7)	63.8 (50.1–76.0)	0.554 (0.424–0.684)	0.127
	CE-CT	0.316	77.8 (52.4–93.6)	70.0 (53.5–83.4)	87.5 (71.0–96.5)	53.8 (33.4–73.4)	72.4 (59.1–83.3)	0.739 (0.617–0.861)	
	CE-PET/CT	0.237	75.0 (58.8–87.3)	55.6 (30.8–78.5)	78.9 (62.7–90.4)	50.0 (27.2–72.8)	68.9 (55.5–80.5)	0.653 (0.517–0.789)	
**AAA**	PET/CT	0.888	61.1 (35.7–82.7)	100 (91.2–100)	85.1 (71.7–93.8)	100 (71.5–100)	87.9 (76.7–95.0)	0.806 (0.689–0.921)	0.056
	CE-CT	0.633	27.8 (9.7–53.5)	97.5 (86.8–99.9)	75.0 (61.1–86.0)	83.3 (35.9–99.6)	75.9 (62.8–86.1)	0.626 (0.517–0.736)	
	CE-PET/CT	0.703	61.1 (35.7–82.7)	95.0 (83.1–99.4)	84.4 (70.5–93.5)	84.6 (54.6–98.1)	84.5 (72.6–92.7)	0.781 (0.659–0.901)	

PET/CT: positron emission tomography/computed tomography; CE: contrast-enhanced; CT: computed tomography; CI: confidence interval; AUC-ROC (area under the receiver operating characteristic curve); NPV: negative predictive value; PPV: positive predictive value; MAA, mycotic aortic aneurysm; IAA, inflammatory aortic aneurysm; AAA, abdominal aortic aneurysm; *: P-value from testing the equality of AUC-ROC across the three imaging methods.

### Imaging characteristics

Relevance of imaging characteristics in PET/CT and CE-CT for diagnosis of MAA, IAA and AAA combined for both readers are displayed in Table 3 (individual data for both readers in S2 Table).

**Table 3:** Relevance of imaging characteristics in PET/CT and CE-CT for diagnosis of MAA, IAA and AAA—combined for both readers.

	MAA	no MAA	p	IAA	no IAA	p	AAA	no AAA	p
**PET/CT**									
SUVmax (median (IQR))	5.4 (4.4–7.2)	3.9 (2.9–5.7)	0.041**	5.6 (3.9–11.2)	4.5 (2.9–5.8)	0.028**	2.9 (2.3–3.9)	5.5 (4.3–8.5)	<0.001**
SUVratio BP (median (IQR))	3.5 (2.5–4.5)	2.7 (1.9–4.1)	0.115	4.1 (2.9–6.2)	2.6 (1.8–3.6)	0.001**	1.9 (1.3–2.1)	3.7 (2.7–4.9)	<0.001**
SUVratio liver (median (IQR))	2.3 (1.9–3.5)	2.3 (1.5–3.1)	0.282	3.1 (2.4–4.6)	1.9 (1.5–2.6)	<0.001**	1.5 (1.2–1.8)	2.6 (2.1–3.5)	<0.001**
wall thickening (n/n (%))*	20/22 (91)	25/36 (69)	0.053	15/18 (83)	30/40 (75)	0.367	10/18 (56)	35/40 (88)	0.011**
dorsal sparing (n/n (%))*	3/22 (14)	13/36 (36)	0.057	9/18 (50)	7/40 (18)	0.014**	4/18 (22)	12/40 (30)	0.391
**CE-CT**									
fat stranding (n/n (%))*	20/22 (91)	19/36 (53)	0.002**	13/18 (72)	26/40 (65)	0.411	6/18 (33)	33/40 (83)	<0.001**
dorsal sparing (n/n (%))*	6/22 (27)	13/36 (36)	0.345	9/18 (50)	10/40 (25)	0.059	4/18 (22)	15/40 (38)	0.201
fluid collection (n/n (%))*	10/22 (45)	5/36 (14)	0.010**	3/18 (17)	12/40 (30)	0.230	2/18 (11)	13/40 (33)	0.077
contrast enhancement (n/n (%))*	10/22 (45)	13/36 (36)	0.333	8/18 (44)	15/40 (38)	0.414	5/18 (28)	18/40 (30)	0.171
wall thickening y/n (n/n (%))*	20/22 (91)	28/36 (78)	0.179	17/18 (94)	31/40 (78)	0.111	11/18 (61)	37/20 (93)	0.007**
wall									
wall thickening (mm (IQR))	9 (7–12)	9 (5–12)	0.596	12 (9–13)	8 (5–10)	0.023**	6 (2–8)	10 (8–13)	0.004**
	**MAA**	**AAA**	**p**	**IAA**	**AAA**	**p**	**MAA**	**IAA**	**p**
**PET/CT**									
SUVmax (median (IQR))	5.4 (4.4–7.2)	2.9 (2.3–3.9)	<0.001**	5.6 (3.9–11.2)	2.9 (2.3–3.9)	<0.001**	5.4 (4.4–7.2)	5.6 (3.9–11.2)	0.77
SUVratio BP (median (IQR))	3.5 (2.5–4.5)	1.9 (1.3–2.1)	<0.001**	4.1 (2.9–6.2)	1.9 (1.3–2.1)	<0.001**	3.5 (2.5–4.5)	4.1 (2.9–6.2)	0.21
SUVratio liver (median (IQR))	2.3 (1.9–3.5)	1.5 (1.2–1.8)	<0.001**	3.1 (2.4–4.6)	1.5 (1.2–1.8)	<0.001**	2.3 (1.9–3.5)	3.1 (2.4–4.6)	0.11
wall thickening (n/n (%))*	20/22 (91)	10/18 (56)	0.025**	15/18 (83)	10/18 (56)	0.146	20/22 (91)	15/18 (83)	0.64
dorsal sparing (n/n (%))*	3/22 (14)	4/18 (22)	0.680	9/18 (50)	4/18 (22)	0.164	3/18 (17)	9/18 (50)	0.018**
**CE-CT**									
fat stranding (n/n (%))*	20/22 (91)	6/18 (50)	<0.001**	13/18 (72)	6/18 (50)	0.044**	20/22 (91)	13/18 (72)	0.21
dorsal sparing (n/n (%))*	6/22 (27)	4/18 (22)	1.0	9/18 (50)	4/18 (22)	0.164	6/22 (27)	9/18 (50)	0.19
fluid collection (n/n (%))*	10/22 (45)	2/18 (11)	0.035**	3/18 (17)	2/18 (11)	1.0	10/22 (45)	3/18 (17)	0.090
contrast enhancement (n/n (%))*	10/22 (45)	5/18 (28)	0.332	8/18 (44)	5/18 (28)	0.489	10/22 (45)	8/18 (44)	1.0
wall thickening y/n (n/n (%))*	20/22 (91)	11/18 (61)	0.053	17/18 (94)	11/18 (61)	0.041**	20/22 (91)	17/18 (94)	1.0
wall									
wall thickening (mm (IQR))	9 (7–12)	6 (2–8)	0.026**	12 (9–13)	6 (2–8)	0.005**	9 (7–12)	12 (9–13)	0.19

PET/CT: positron emission tomography/computed tomography; CE: contrast-enhanced; CT: computed tomography; MAA, mycotic aortic aneurysm; IAA: inflammatory aortic aneurysm; AAA: abdominal aortic aneurysm IQR: interquartile range; SUVmax: maximum standardized uptake value; BP: blood pool; y/n: yes/no; * referring to the number of patients with a given imaging characteristic in a group of patients with the respective diagnosis, i.e. fifth line, second column: in 22 datasets with MAA (11 for each reader), readers determined the imaging characteristic “wall thickening” in 20 cases (91%); ** indicates statistical significance.

Briefly, metabolic acitivity in PET/CT (i.e., SUVmax, SUVratio BP, and SUVratio liver) in non-infected, non-inflammatory AAA was significantly lower as compared to MAA and IAA, while it was significantly higher in all IAA compared to MAA and AAA. In PET/CT, significantly lower rates of wall thickening were detected in AAA and dorsal sparing was significantly more frequent in IAA (Fig 1). In CE-CT, significantly lower rates of fat stranding were detected by both readers in AAA, while significantly higher rates of fat stranding and fluid collections were detected in MAA. Furthermore, the wall thickness of AAA was significantly lower as compared to MAA and IAA, and significantly higher in IAA as compared to MAA and AAA (Figs 1–5).

## Discussion

Differentiating MAA, IAA and AAA by imaging is known to be cumbersome [4], as underlined by a relatively high interrater variability in all imaging modalities of the present study, and by trends toward relevant differences of diagnostic accuracy between readers. However, we could determine imaging characteristics and differences in diagnostic accuracy, which may be helpful in discriminating the three identities and in increasing the diagnostic performance of imaging.

Namely, we observed higher diagnostic accuracy of PET/CT over CE-CT in differentiating non-infected, non-inflammatory AAA from MAA and IAA. Furthermore, imaging characteristics of PET/CT and CE-CT may help to differentiate between MAA and IAA, as very high metabolic activity and dorsal sparing of metabolic activity in PET/CT and wall thickening in CE-CT are indicative for an IAA, while fat stranding and fluid collections in CE-CT are indicative of a MAA. Finally, low metabolic acitivity and missing wall thickness in PET/CT, and low rates of fat stranding and missing wall thickness in CE-CT are indicative for non-infected, non-inflammatory AAA.

An initial report comparing diagnostic accuracy of CE-CT and PET/CT in MAA was hampered by numerous false positive findings due to inflammatory aneurysms [10]. To the best of our knowledge, our study is the first to comprehensively compare diagnostic accuracy of PET/CT and CE-CT and assess imaging characteristics for the diagnosis of MAA, IAA and AAA.

IAA may present with a wide spectrum of inflammation [12], making the diagnosis difficult, and none of the presented imaging modalities showed a significantly higher accuracy in diagnosing IAA. Aortic wall thickening, sparing the posterior wall (also known as “dorsal sparing” or “mantle sign”) is a known imaging characteristic of IAA [2, 9], which is however, also encountered in MAA [4], as confirmed for CE-CT and for PET/CT in our presented data (Table 3). Furthermore, contrast enhancement of the thickened aortic wall is a previously described characteristic radiologic imaging feature in CE-CT for IAA, which could not be confirmed by our data, possibly owing to the small sample size. However, in PET/CT the thickened aortic wall showed significantly higher metabolic activity, presumably a new and more sensitive imaging finding as contrast enhancement in CT.

For MAA, known imaging characteristic in CE-CT [2] are perianeurysmal gas formation, fat stranding, and periaortic soft tissue masses with or without contrast enhancement, while focally increased FDG uptake is indicative of an MAA in PET/CT [10]. We could confirm that fat stranding and fluid collections in CE-CT are indicative of an MAA, while all other imaging characteristics did not significantly differ in our study. Furthermore, the values for diagnostic accuracy in the present study were somewhat lower as previously reported in the literature [10]. Both of which, may partly be attributed to the present study design (as further discussed in the limitations section).

PET/CT displayed higher diagnostic accuracy over CE-CT in differentiating non-infected, non-inflammatory AAA from MAA and IAA, which may be considered an important new finding for future imaging in patients with abdominal aneurysms.

However, previous studies have also described increased metabolic activity in aneurysms in general [13] and focally increased FDG uptake in the aneurysmal wall preceding rupture of AAA [14]. The latter issue may have led to some of the false findings in the present study, and it appears of utmost importance to be aware, that the differential diagnoses of focal FDG-activity in an abdominal aneurysm include IAA, MAA as well as “symptomatic non-ruptured AAA in a hemodynamically unstable patient” (previously called”pre-rupture”) (Fig 4).

In summary, differentiating MAA, IAA and AAA with PET/CT and CE-CT is difficult, and neither imaging method may be considered superior. Certain imaging features are significantly associated with a specific aneurysm etiology. Therefore we believe, that a combined imaging approach consisting of CE-PET/CT may be helpful to establish the diagnosis of MAA, IAA and AAA in clinical routine as an adjunct to the patient’s clinical features and laboratory findings [4].

### Limitations

We may have introduced a selection bias, as in all our study subjects a vascular abnormality was somehow expected or considered possible in the patient’s clinical history and/or written PET/CT report. The latter may have resulted in a low number of normal cases, involving a probable underestimation of the diagnostic accuracy for normal non-infected, non-inflammatory AAA and a potential overestimation of the diagnostic accuracy for MAA and AAA owing to a detection bias.

Secondly, the attempt to differentiate two—in terms of appearance in medical imaging similar—diagnoses (i.e., MAA and IAA), we may have underestimated the diagnostic accuracy of the methods as compared to previous reports [9, 10].

Thirdly, the prevalence of MAA and IAA was supposedly higher than in a normal clinical setting, which, according to the Bayesian theorem, leads to an overestimation of the PPV and an underestimation of the NPV.

Fourthly, we did not specifically analyse further types of distribution patterns of FDG (apart from dorsal sparing) to differentiate between MAA and IAA, which may be an interesting approach in upcoming studies with larger patient populations.

Finally, we did not adjust p-values for multiple testing, and therefore false positive results cannot be excluded with certainty.

## Conclusion

Specific imaging characteristics of PET/CT and CE-CT may be helpful in differentiating between MAA, IAA, and non-infected, non-inflammatory AAA. Combined CE-PET/CT imaging might represent the optimal imaging approach for this purpose.

## Supporting information

S1 Table Relevance of imaging characteristics in PET/CT and CE-CT for diagnosis of MAA, IAA and AAA—individual data for both readers. (DOCX)

S2 Table Relevance of imaging characteristics in PET/CT and CE-CT for diagnosis of MAA, IAA and AAA—individual data for both readers. (DOCX)

S1 Data (XLSX)
