# Supplementary material for: Imaging characteristics and diagnostic accuracy of FDG-PET/CT, contrast enhanced CT and combined imaging in patients with suspected mycotic or inflammatory abdominal aortic aneurysms
Source: PLoS One. 2022 Aug 9;17(8):e0272772. doi: 10.1371/journal.pone.0272772 (PMC9362916; doi:10.1371/journal.pone.0272772)
Supplement: S1 Table — (DOCX) [file pone.0272772.s001.docx]

**Supplemental Table 1.** Diagnostic accuracy of PET/CT, CE-CT, combined CE-PET/CT for diagnosis of MAA, IAA and AAA

|  | | | **Sensitivity** | **Specificity** | **NPV** | **PPV** | **Accuracy** |
| --- | --- | --- | --- | --- | --- | --- | --- |
|  |  |  | % (95% CI) | % (95% CI) | % (95% CI) | % (95% CI) | % (95% CI) |
| **MAA** | reader 1 | PET/CT | 72.7 (39.0-94.0) | 38.9 (17.3-64.3) | 70.0 (34.8-93.3) | 42.1 (20.3-66.5) | 51.7 (32.5-70.6) |
|  |  | CE-CT | 63.6 (30.8-89.1) | 72.2 (46.5-90.3) | 76.5 (50.1-93.2) | 58.3 (27.7-84.8) | 68.9 (49.2-84.7) |
|  |  | CE-PET/CT | 63.6 (30.8-89.1) | 72.2 (46.5-90.3) | 76.5 (50.1-93.2) | 58.3 (27.7-84.8) | 68.9 (49.2-84.7) |
|  | reader 2 | PET/CT | 72.7 (39.0-94.0) | 72.2 (46.5-90.3) | 81.3 (54.4-96.0) | 61.5 (31.6-86.1) | 72.4 (52.8-87.3) |
|  |  | CE-CT | 72.7 (39.0-94.0) | 66.7 (41.0-86.7) | 80.0 (51.9-95.7) | 57.1 (28.9-82.3) | 68.9 (49.2-84.7) |
|  |  | CE-PET/CT | 90.9 (58.7-99.8) | 83.3 (58.6-96.4) | 93.8 (69.8-99.8) | 76.9 (46.2-95.0) | 86.2 (68.3-96.1) |
| **IAA** | reader 1 | PET/CT | 11.1 (0.3-48.2) | 80.0 (56.3-94.3) | 66.7 (44.7-84.4) | 20.0 (0.5-71.6) | 58.6 (38.9-76.5) |
|  |  | CE-CT | 88.9 (51.8-99.7) | 65.0 (40.8-84.6) | 92.9 (66.1-99.8) | 53.3 (26.6-78.7) | 72.4 (52.8-87.3) |
|  |  | CE-PET/CT | 33.3 (7.5-70.1) | 65.0 (40.8-84.6) | 68.4 (43.4-87.4) | 30.0 (6.7-65.2) | 55.2 (35.7-73.6) |
|  | reader 2 | PET/CT | 55.6 (21.2-86.3) | 75.0 (50.9-91.3) | 78.9 (54.4-93.9) | 50.0 (18.7-81.3) | 68.9 (49.2-84.7) |
|  |  | CE-CT | 66.7 (29.9-92.5) | 75.0 (50.9-91.3) | 83.3 (58.6-96.4) | 54.5 (23.4-83.3) | 72.4 (52.8-87.3) |
|  |  | CE-PET/CT | 77.8 (40.0-97.2) | 85.0 (62.1-96.8) | 89.5 (66.9-98.7) | 70.0 (34.8-93.3) | 82.8 (64.2-94.2) |
| **AAA** | reader 1 | PET/CT | 55.6 (21.2-86.3) | 100 (83.2-100) | 83.3 (62.6-95.3) | 100 (47.8-100) | 86.2 (68.3-96.1) |
|  |  | CE-CT | 22.2 (2.8-60.0) | 100 (83.2-100) | 74.1 (53.7-88.9) | 100 (15.8-100) | 75.9 (56.5-89.7) |
|  |  | CE-PET/CT | 55.6 (21.2-86.3) | 90.0 (68.3-98.8) | 81.8 (59.7-94.8) | 71.4 (29.0-96.3) | 79.3 (60.3-92.0) |
|  | reader 2 | PET/CT | 66.7 (29.9-92.5) | 100 (83.2-100) | 87.0 (66.4-97.2) | 100 (54.1-100) | 89.7 (72.7-97.8) |
|  |  | CE-CT | 33.3 (7.5-70.1) | 95.0 (75.1-99.9) | 76.0 (54.9-90.6) | 75.0 (19.4-99.4) | 75.9 (56.5-89.7) |
|  |  | CE-PET/CT | 66.7 (29.9-92.5) | 100 (83.2-100) | 87.0 (66.4-97.2) | 100 (54.1-100) | 89.7 (72.7-97.8) |

PET/CT: positron emission tomography/computed tomography; CE: contrast-enhanced; CT: computed tomography; CI: confidence interval; SUV_max_.: maximum standardized uptake value; NPV: negative predictive value; PPV: positive predictive value; MAA, mycotic aortic aneurysm; IAA, inflammatory aortic aneurysm; AAA, abdominal aortic aneurysm
