# Supplementary material for: Imaging characteristics and diagnostic accuracy of FDG-PET/CT, contrast enhanced CT and combined imaging in patients with suspected mycotic or inflammatory abdominal aortic aneurysms
Source: PLoS One. 2022 Aug 9;17(8):e0272772. doi: 10.1371/journal.pone.0272772 (PMC9362916; doi:10.1371/journal.pone.0272772)
Supplement: S2 Table — (DOCX) [file pone.0272772.s002.docx]

**Supplemental Table 2.** Relevance of imaging characteristics in PET/CT and CE-CT for diagnosis of MAA, IAA and AAA

| **PET/CT** | **MAA** | **no MAA** | **p** | **IAA** | **no IAA** | **p** | **AAA** | **no AAA** | **p** |
| --- | --- | --- | --- | --- | --- | --- | --- | --- | --- |
| **Reader 1:** |  |  |  |  |  |  |  |  |  |
| SUVmax (median (IQR)) | 5.8 (4.4-7.2) | 3.9 (2.9-5.8) | 0.138 | 5.6 (3.9-11.2) | 4.5 (2.7-5.9) | 0.118 | 2.9 (2.3-3.9) | 5.7 (4.3-9.3) | <0.001** |
| SUVratio BP (median (IQR)) | 3.5 (2.5-4.5) | 2.6 (1.9-4.4) | 0.243 | 4.4 (2.9-6.2) | 2.6 (1.8-3.8) | 0.023** | 1.9 (1.3-2.0) | 3.9 (2.6-5.3) | <0.001** |
| SUVratio liver (median (IQR)) | 2.6 (2.1-3.6) | 2.4 (1.5-3.2) | 0.500 | 3.2 (2.5-5.4) | 2.1 (1.4-2.6) | 0.013** | 1.5 (1.2-1.8) | 2.6 (2.2-4.5) | <0.001** |
| wall thickening (n/n (%))* | 11/11 (100) | 13/18 (72) | 0.072 | 7/9 (78) | 17/20 (85) | 0.502 | 6/9 (67) | 18/20 (90) | 0.157 |
| dorsal sparing (n/n (%))* | 2/11 (18) | 6/18 (33) | 0.330 | 4/9 (44) | 4/20 (20) | 0.180 | 2/9 (22) | 6/20 (30) | 0.517 |
| **Reader 2:** |  |  |  |  |  |  |  |  |  |
| SUVmax (median (IQR)) | 5.1 (4.4-7.2) | 3.8 (3.0-5.6) | 0.157 | 5.0 (4.0-9.2) | 4.5 (2.9-5.6) | 0.149 | 3.0 (2.3-3.3) | 5.1 (4.2-8.2) | 0.002** |
| SUVratio BP (median (IQR)) | 3.4 (2.1-4.7) | 2.8 (1.8-4.0) | 0.281 | 4.0 (2.9-5.1) | 2.3 (1.7-3.5) | 0.034** | 1.8 (1.3-2.1) | 3.5 (2.7-4.9) | <0.001** |
| SUVratio liver (median (IQR)) | 2.2 (1.8-3.5) | 2.1 (1.5-3.1) | 0.419 | 3.1 (2.3-3.4) | 1.8 (1.5-2.6) | 0.030** | 1.5 (1.3-1.8) | 2.4 (1.9-3.5) | 0.002** |
| wall thickening (n/n (%))* | 9/11 (82) | 12/18 (67) | 0.330 | 8/9 (89) | 13/20 (65) | 0.192 | 4/9 (44) | 17/20 (85) | 0.037** |
| dorsal sparing (n/n (%))* | 1/11 (9) | 7/18 (39) | 0.092 | 5/9 (56) | 3/20 (15) | 0.037** | 2/9 (22) | 6/20 (30) | 0.517 |
| **CE-CT** | **MAA** | **no MAA** | **p** | **IAA** | **no IAA** | **p** | **AAA** | **no AAA** | **p** |
| **Reader 1:** |  |  |  |  |  |  |  |  |  |
| fat stranding (n/n (%))* | 10/11 (91) | 8/18 (44) | 0.015** | 5/9 (56) | 13/20 (65) | 0.466 | 3/9 (33) | 15/20 (75) | 0.043** |
| dorsal sparing (n/n (%))* | 1/11 (9) | 6/18 (33) | 0.151 | 4/9 (44) | 3/20 (15) | 0.108 | 2/9 (22) | 5/20 (25) | 0.631 |
| fluid collection (n/n (%))* | 5/11 (45) | 2/18 (11) | 0.051 | 1/9 (11) | 6/20 (30) | 0.273 | 1/9 (11) | 6/20 (30) | 0.273 |
| contrast enhancement (n/n (%))* | 4/11 (36) | 3/18 (17) | 0.223 | 2/9 (22) | 5/20 (25) | 0.631 | 1/9 (11) | 6/20 (30) | 0.273 |
| wall thickening y/n (n/n (%))*  wall | 11/11 (100) | 14/18 (78) | 0.129 | 8/9 (89) | 17/20 (85) | 0.636 | 6/9 (67) | 19/20 (95) | 0.076 |
| wall thickening (mm (IQR)) | 9 (7-12) | 10 (5-12) | 0.946 | 12 (10-13) | 8 (6-10) | 0.081 | 6 (4-10) | 10 (8-13) | 0.069 |
| **Reader 2:** |  |  |  |  |  |  |  |  |  |
| fat stranding (n/n (%))* | 10/11 (91) | 11/18 (61) | 0.092 | 8/9 (89) | 13/20 (65) | 0.192 | 3/9 (33) | 18/20 (90) | 0.004** |
| dorsal sparing (n/n (%))* | 5/11 (45) | 7/18 (39) | 1.000 | 5/9 (56) | 7/20 (35) | 0.263 | 2/9 (22) | 10/20 (50) | 0.160 |
| fluid collection (n/n (%))* | 5/11 (45) | 3/18 (17) | 0.106 | 2/9 (22) | 6/20 (30) | 0.517 | 1/9 (11) | 7/20 (35) | 0.192 |
| contrast enhancement (n/n (%))* | 6/11 (55) | 10/18 (56) | 0.628 | 6/9 (67) | 10/20 (50) | 0.336 | 4/9 (44) | 12/20 (60) | 0.353 |
| wall thickening y/n (n/n (%))*  wall | 9/11 (82) | 14/18 (78) | 0.592 | 9/9 (100) | 14/20 (70) | 0.082 | 5/9 (56) | 18/20 (90) | 0.056 |
| wall thickening (mm (IQR)) | 10 (4-13) | 8 (6-12) | 0.499 | 11 (9-12) | 8 (4-11) | 0.155 | 6 (2-8) | 10 (8-13) | 0.031** |

PET/CT: positron emission tomography/computed tomography; CE: contrast-enhanced; CT: computed tomography; MAA, mycotic aortic aneurysm; IAA: inflammatory aortic aneurysm; AAA: abdominal aortic aneurysm; IQR: 95% interquartile range; SUVmax: maximum standardized uptake value; BP: blood pool; y/n: yes/no; * referring to the number of patients with a given imaging characteristic in a group of patients with the respective diagnosis, i.e. sixth line, second column: in 11 patients with MAA, reader 1 determined the imaging characteristic “wall thickening” in 11 patients (100%); ** indicates statistical significance.
